# Supplementary material for: The interactome of intact mitochondria by cross-linking mass spectrometry provides evidence for coexisting respiratory supercomplexes
Source: Mol Cell Proteomics. 2017 Dec 8;17(2):216–32. doi: 10.1074/mcp.RA117.000470 (PMC5795388; doi:10.1074/mcp.RA117.000470)
Supplement: Supplemental Data [file supp_RA117.000470_133922_0_supp_23978_fzffwf.zip › spectra_annotation/mito_DR_spectra_annotation/4-2-37-1-11-1.pdf]

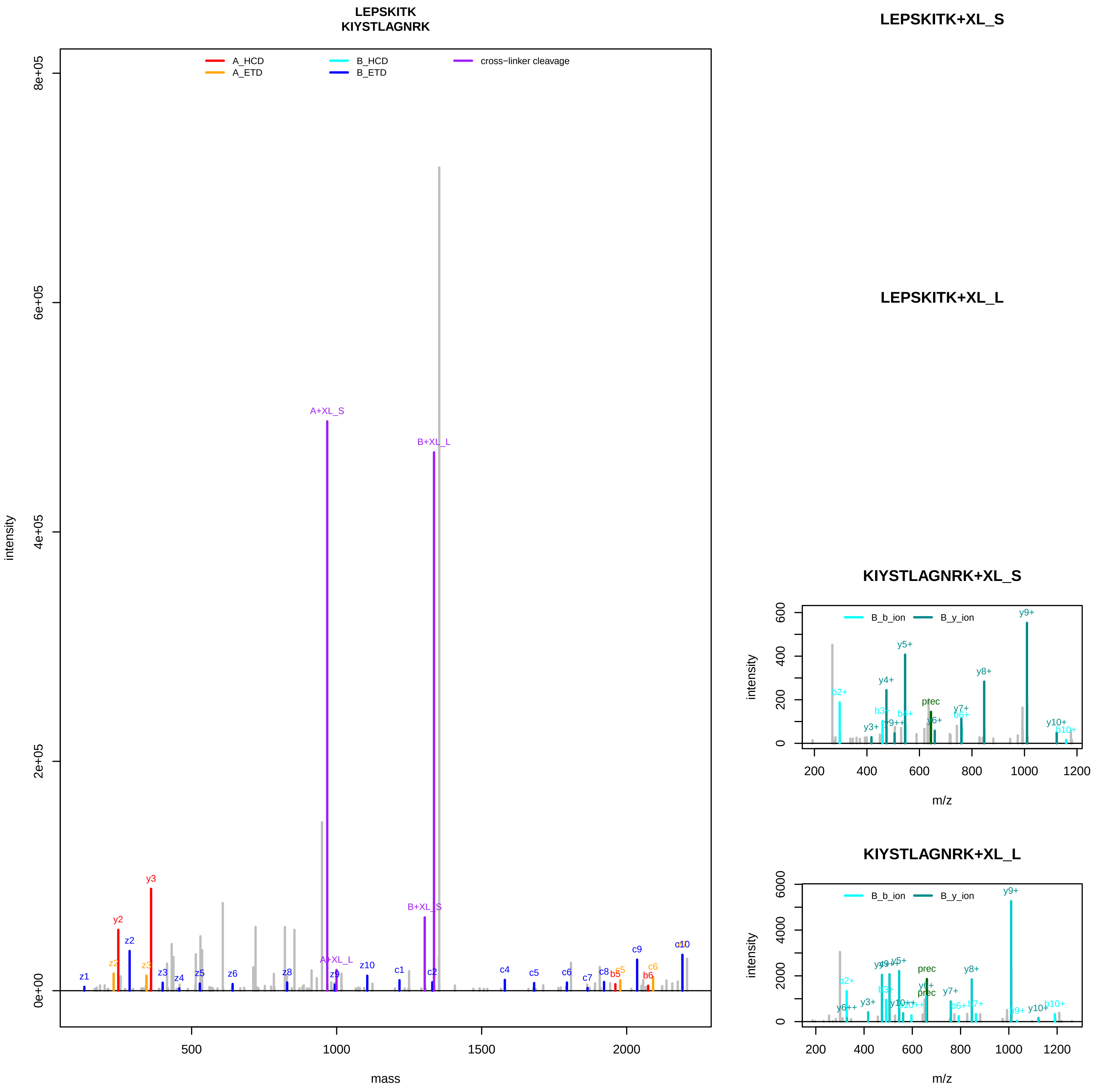
LEPSKITK
KIYSTLAGNRK
A_HCD
A_ETD
B_HCD
B_ETD
cross−linker cleavage
A+XL_S
B+XL_L
B+XL_S
A+XL_L
y2
y3
z1
z2
z3
z4
z5
z6
z8
z9
z10
c1
c2
c4
c5
c6
c7
c8
c9
c10
b5
b6
mass
intensity
0e+00
2e+05
4e+05
6e+05
8e+05
500
1000
1500
2000
LEPSKITK+XL_S
LEPSKITK+XL_L
KIYSTLAGNRK+XL_S
B_b_ion
B_y_ion
y9+
y5+
y4+
y8+
prec
y10+
b10+
b2+
y7+
y6+
y3+
m/z
intensity
KIYSTLAGNRK+XL_L
B_b_ion
B_y_ion
y9+
y8+
prec
y7+
b2+
y3+
b10+
y10+
m/z
intensity
